# Sensing the future of bio-informational engineering

**DOI:** 10.1038/s41467-020-20764-2

**Published:** 2021-01-15

**Authors:** Thomas A. Dixon, Thomas C. Williams, Isak S. Pretorius

**Affiliations:** 1 Department of Modern History, Politics and International Relations, Macquarie University, Sydney, NSW 2109 Australia; 2 Department of Molecular Sciences, Macquarie University, Sydney, NSW 2109 Australia; 3 ARC Centre of Excellence in Synthetic Biology, Macquarie University, Sydney, NSW 2109 Australia; 4 Chancellery, Macquarie University, Sydney, NSW 2109 Australia

**Keywords:** Synthetic biology, Interdisciplinary studies

## Abstract

The practices of synthetic biology are being integrated into ‘multiscale’ designs enabling two-way communication across organic and inorganic information substrates in biological, digital and cyber-physical system integrations. Novel applications of ‘bio-informational’ engineering will arise in environmental monitoring, precision agriculture, precision medicine and next-generation biomanufacturing. Potential developments include sentinel plants for environmental monitoring and autonomous bioreactors that respond to biosensor signaling. As bio-informational understanding progresses, both natural and engineered biological systems will need to be reimagined as cyber-physical architectures. We propose that a multiple length scale taxonomy will assist in rationalizing and enabling this transformative development in engineering biology.

## Abstract (web summary)

Synthetic biology engineering principles enable two-way communication between living and inanimate substrates. Here the authors consider the development of this bio-informational exchange and propose cyber-physical architectures and applications.

## Introduction

**Fig. 1:**
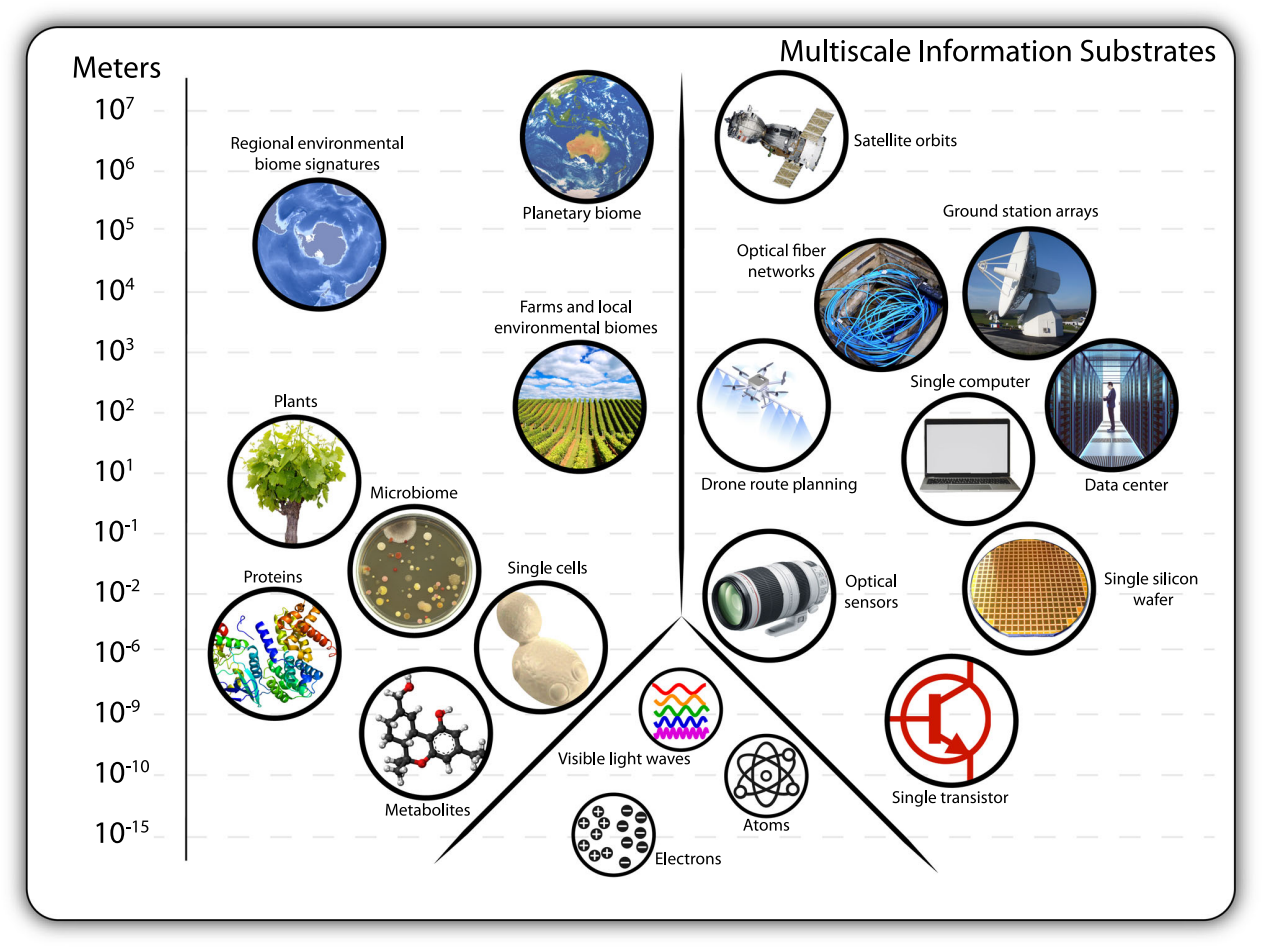
Multiple length scale engineering across organic and inorganic information substrates. Engineering information communication across substrates interfaces a diverse array of systems over multiple length scales. Bio-informational system integrations operate across 10^−15^ m through to 10^7^ m. To date, this process has primarily involved the collection of biological metadata via digital means. Developments in optogenetics and bioelectrochemistry suggest multiscale engineering may become a two-way communication channel.

Synthetic biology’s multiscale approach to biosensing enables real-time data capture from many different types of environment—built, natural, and living. Biological devices are being reimagined as advanced cyber-physical systems through their integration with digital and mechanical length scales. Engineering biology is moving beyond mechanical biomimicry^1^ and next-generation biological devices are beginning to make use of life’s multiscale information architectures^2^. We use the term multiscale here to describe the integration of different engineering length scales (10^*x*^ metres) into one design and solution (Fig. 1). This perspective is primarily focused on the opportunities and challenges arising from engineering across length scales that bootstrap biological functionality—that is, electrical, chemical, and optical scales of existence. We call this ‘bio-informational engineering’ because biological devices are finely tuned sensor arrays optimized for translating non-traditional information into the digital world. Importantly, we anticipate that the global research and development response to the COVID-19 pandemic will accelerate the advance of bio-informational engineering across the coming years.

### Multiscale biosensing

Biological systems predominantly operate using biochemicals for information storage (nucleic acids), energy processing (metabolism), and growth (biomass). However, these naturally evolved systems are poised for integration with engineered electronics and electromagnetic information processing systems using a variety of mechanisms. For example, cellular metabolism relies heavily on reduction/oxidation (redox) reactions that involve electron transfer. Electrons are transferred between membrane-bound proteins during respiration and photosynthesis (redox), nerve cells transfer information using electrochemical action potentials (ionic current), and electrical current can be conducted between bacterial cells using protein nanowires (molecular conductivity)^3^. Life also frequently transfers information using forms of electromagnetic radiation, such as luminescence (chemical to light conversion), fluorescence (light to light conversion), and iridescence (structural light diffraction). Life has evolved highly complex systems, such as photosynthesis, these systems can convert energy, and therefore information, between optical and chemical forms.

Interfacing human digital and electrical systems to measure and control biology can be achieved by converting cellular signals, such as metabolite and protein concentrations into electrical or electromagnetic radiation signals using synthetic biology tools, such as biosensors, optogenetics, electrogenetics, and established concepts in bioelectrochemistry. The convergence of biological and digital information systems promises to facilitate unprecedented insight into, and control of, biological systems ranging from single cells, multi-cellular organisms, and entire ecosystems. Engineering organisms that respond in a well-characterized manner to optical signalling will enable digital-to-biology control loops with low latency. Optogenetics is a rapidly maturing field that enables precise control of gene expression in response to specific wavelengths of light^4^. Bioelectrical research is another sub-discipline disabling barriers to bio-informational engineering^5^. This field is opening up novel ways to control cell behaviours through electrical and electrochemical means. Integrating biology with two-way digital control loops further expands the vast design space of biological devices. Two-way bio-informational functionality could enable organism-to-organism signalling through an Internet of Biological Things (IoBT) and satellite communications infrastructure, disconnecting organism communication from time–space constraints. Networks of biosensors and biological devices could be distributed across disparate geographies connected through an always-on information exchange. This could revolutionize agricultural productivity, human health, environmental monitoring, and next-generation robotic architectures. In this Perspective, we offer an overview of the current state of synthetic biology research in biosensors, optogenetic signalling, and bioelectrical interfacing. We also offer an outline of key challenges in these areas that need to be overcome in order to rationalize two-way bio-informational communication (Table 1). We then outline some promising future directions for multiscale bio-informational research.

**Table 1:** Development prospects for bio-informational engineering.

	Current state	Key problems	Future directions
Biosensors	Biological detection and response of target molecules with high specificity and sensitivity.	Engineering ligand-binding domains and standardizing biosensor architectures.	Rationalizing a translation pathway for information from a biological to a digital format.
Optogenetics	The use of specific wavelengths of light to control proteins in living cells.	Multiplexing, signal insulation, and biomass absorbance.	Engineered orthogonal light responsive proteins and refactored host organisms.
Bioelectrochemistry	Direct measurement and control of various biological signals and processes via electrical signalling.	Matching biological circuitry complexity to electronic inputs and decreasing information loss between electronic and biological mediums.	Integrating biological systems with electronics and computing architectures.
Internet of Biological Things	Networked cyber-physical and optoelectronic systems that harvest, analyse, model, sequence, and synthesize organic information substrates.	Lowering bio-informational signal loss during substrate conversion and developing standard bio-information structures. Engineering robust and widespread two-way biological to electronic communication.	Networked natural and engineered living systems integrating satellite architectures and low-latency signal conversion between organic and inorganic information substrates. Two-way biological-electronic communication mediated by Artificial Intelligence (AI).

### Biosensors

Biosensors are a class of genetically encoded biological molecules, proteins, cells, or cell consortia that can be used to detect and respond to target ligands with high specificity and sensitivity^6–9^. Biosensors can be comprised of protein, RNA, or DNA structures, depending on the application and its requirements, and can be either naked molecules, or functional within cells and their native regulatory components. Biosensors can also be comprised of whole cells^10^, synthetic co-cultures^11^, or non-genetically encoded biosensors interfaced with cells^12^. In genetically encoded biosensors such as nucleic acids and protein, detection and signal actuation domains are typically connected so that the binding of each target molecule to a sensor domain can initiate signal output by inducing a conformational change in the sensor. The range of molecules that biosensors can detect is incredibly diverse, representing the broad capabilities of biochemistry to interact at the atomic, molecular, organic/inorganic, cellular, and multicellular scales. Depending on the biosensor architecture, output signals could be gene expression, enzyme activity, light, fluorescence, or electron release^6^. Due to these broad capabilities, target specificity, and natural interface with living systems, biosensors are one of the keys to the future of bio-informational engineering, which will enable two-way communication between human digital technology and biological processes.

Biosensors have proliferated in the field of synthetic biology, which aims to enable fine level control and understanding of biology using engineering principles^13^. A large proportion of early synthetic biology research was focused on controlling the strength and timing of gene expression in biological circuits and metabolic pathways. This was typically achieved by characterizing transcription factor promoter pairs, and tuning their interactions. Recently, synthetic biology has co-opted biosensors to enable dynamic and autonomous control of gene expression in response to endogenous cellular conditions, such as metabolite or protein concentrations. This is achieved by having key circuit or pathway genes expressed in response to fluctuating endogenous metabolite or protein levels so that optimal synthetic protein concentrations can be automatically adjusted^14,15^. Similar ‘closed loop’ systems have been engineered in human cells that produce insulin in response to glucose levels^16^. In general, biosensors have also become important tools in synthetic biology efforts focused on mammalian cells. For example, synthetic notch receptors were developed with a fully modular transmembrane domain that can be connected to extracellular ligand-binding domains (LBDs) and intracellular transcription factors^17^. Upon ligand binding, the intracellular domain is proteolytically cleaved from the transmembrane domain, and can actuate intracellular outputs such as transcription. This system was exploited to engineer diverse outputs such as cell patterning, differentiation, cell–cell communication, and computational logic functions in response to diverse environmental stimuli. Another prominent use of biosensors in synthetic biology has been to enable ultra-high throughput screening of genetic variants for their capacity to produce a metabolite^18,19^. In this scenario, the biosensor detects the intracellular concentration of the target metabolite, typically a valuable fuel, chemical, or pharmaceutical, and outputs a selection/survival function, such as fluorescence in response. This enables the selection of the highest metabolite producers from a pool of random genetic variants. Due to the high value of these two applications of biosensors in synthetic biology, the field has begun to solve some of the most significant challenges in biosensor technology.

### Challenges and future directions

#### Engineered domains and standardized biosensor architectures

**Fig. 2:**
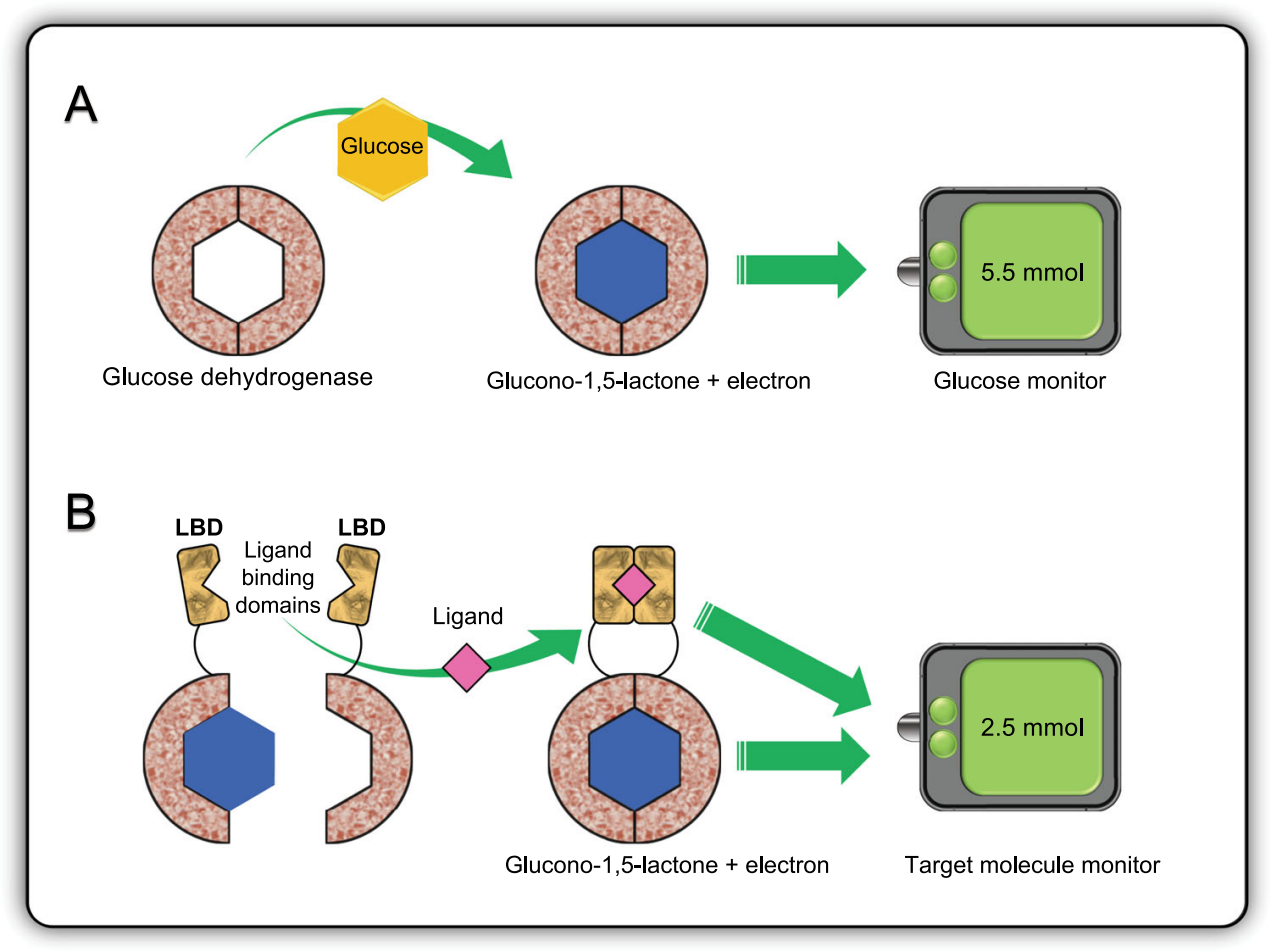
Engineering ligand-binding domains creates opportunities for translating the biosensing of target molecules into digital signals. (**A**) Pyrroloquinoline quinone glucose dehydrogenase (GDH) releases an electron in the presence of glucose, which can be detected using an electrode in a blood-glucose monitor. (**B**) Split GDH fused to ligand-binding domains (LBDs) for another molecule can be used as a generic biosensor, as the GDH domains can only co-localize to release an electron when the fused LBDs bind to a molecule of interest.

There are two major challenges that need to be overcome before wide-scale deployment of biosensors for bio-informational engineering can be realized. The first is a lack of detection domains for every conceivable target biosensor molecule. Although nature has evolved proteins and nucleic acids that bind to numerous molecules, many are not characterized and do not result in the conformational changes necessary to generate a signal output. Although methods have been developed to modularize allosteric transcription factors^20–22^, evolve nucleic acids^23^, select peptides and antibodies^24^, and modify existing protein domains to have signal output functions^25,26^, none of these methods can reliably produce detection domains that are readily interfaced with a signal output biosensor domain. This leads to the second major challenge facing biosensor development, which is the lack of a standardized biosensor architecture. A standardized, modular biosensor architecture would allow for routine integration of signal response, actuation, and output components. There has been significant progress in the standardization of signal outputs such as Fluorescence Resonance Energy Transfer (FRET) pairs that emit a distinct fluorescence signal based on protein proximity. Another example is the use of glucose dehydrogenases that can transfer electrons to an electrode in response to binding glucose molecules (Fig. 2)^27^. Glucose dehydrogenase sensors have been successfully deployed as blood-glucose monitors for diabetics and are the benchmark for current bio-informational engineering technologies. However, interfacing a signal output architecture with signal detection components currently requires the use of protein-binding domains that undergo large conformation shifts in response to target molecule binding, or the splitting of LBDs. Ligand-binding proteins that undergo large conformational shifts are rare relative to desired ligand diversity, and not all ligand-binding proteins can be reliably split such that they co-localize in response to a target ligand. These two limitations have thus far excluded the engineering of modular and standardized biosensor architectures. A technology that enables evolution or selection of structurally complex co-localization LBDs would solve these problems. Such a technology would provide custom-made binding domains that are readily interfaceable with existing FRET, transcriptional, and electronic biosensor architectures for any target molecule.

Biosensors are critical for enabling conversion of biological signals into digital signals that can be used to monitor environments at different length scales. For example, biosensors could be used for real-time monitoring of pollution and environmental contamination, agricultural and cellular production systems, and for tracking of health-related markers in bodily fluids and the gastrointestinal tract. In the future, biosensors will translate information from biological to digital mediums, providing the first step in two-way bio-informational communication and engineering.

### Optogenetics

#### A bright future for bio-informational engineering

Optogenetics is a field that uses specific wavelengths of light to control proteins in living cells. In contrast to traditional forms of chemical modulation of cell physiology, optogenetics facilitates a finer level of spatiotemporal control due to the digital nature of modern light-emitting electronics. The field of optogenetics began in the early 2000s when researchers used light-responsive membrane ion channels to modulate action potentials in neurons^28^. The discovery and utilization of phytochromes and light oxygen voltage (LOV) domain proteins that undergo a conformational change in response to specific wavelengths of light provided a more versatile and modular mechanism for controlling biochemistry with light^4^.

Light-induced conformational shifts can be transduced into other biochemical activities in two main ways (in addition to light-mediated ion channels, which are popular in neural optogenetics)^29,30^. Firstly, in a technique called photocaging, an allosteric enzyme inhibition domain can be fused to a photoisomerizable ‘switch’ domain, which is in turn fused to an enzyme. In the presence of specific light wavelengths, the allosteric inhibition domain can move in response to a change in the photoisomerizable domain to block the active site of the enzyme. This mechanism is not sufficiently modular or generic to have facilitated widespread use since it requires known allosteric domains for given enzymes, requires proteins to function as fusions, and likely needs linker peptide length and flexibility optimization to enable efficient conformational change transduction. The second major mechanism for optogenetics is far more modular as it relies on protein colocalization. The phytochrome and phytochrome interacting factor (PIF) proteins from *Arabidopsis thaliana* use this mechanism, and are one of the predominant tools in optogenetics (for more, please see the OptoBase database)^31^. The PIF can reversibly associate with the phytochrome in response to red or near infrared (NIR) light^32^, providing a means to induce and reverse the co-localization of signal output domains that modify cellular physiology. Given that protein–protein interactions are one of the predominant cellular informational currencies, these mechanisms can be used to optogenetically control a variety of processes. For example, separate transcriptional activation and DNA-binding domains can induce transcription in response to light when they are fused to optogenetic co-localization domains^33^, CRE recombinase can be split such that activity is dependent on light-induced co-localization^34,35^, intracellular signalling cascades can be controlled^36^, and protein localization can be modulated by tethering optogenetic protein interaction domains to organelles, such as the plasma membrane^37^. An emerging application in basic research is the regulation of endogenous proteins encoded from their native genomic context via light-induced antibody binding^38,39^. This circumvents the introduction of confounding variables from genetically manipulating native proteins to achieve optogenetic regulation. Another highly modular option for manipulating gene expression is via the use of photo-regulated CRISPR-dCas9 systems, which can be guided to synthetic or native promoters to induce or repress gene expression in a light-dependent manner^40^.

### Challenges and future directions

#### Multiplexing, signal insulation, and biomass absorbance

One of the major challenges in the future development of optogenetics is expanding the repertoire of photoreceptors to enable wavelength multiplexing. Multiplexing enables more complex functions to be light-encoded in synthetic cells. Much headway has already been made in this area with blue, red, and NIR responsive proteins widely available^4,41^. Another challenge for the implementation of optogenetics in bio-informational engineering is the fact that visible light does not penetrate high cell-density bioreactors or biological tissues sufficiently to reach all cells. This is a feature that has prevented the economically viable use of photosynthetic microorganisms for the production of a range of biological chemicals and fuels. Despite this barrier, optogenetic control of yeast fermentation was recently demonstrated by fine-tuning the timing and expression level of a mitochondria-targeted synthetic pathway for isobutanol production^42^, and for the assembly of a synthetic violacein enzyme cluster^43^. The penetrance of light into deep and dense biological masses could be improved using non-biological engineering solutions involving immersed bioreactor light sources, alternate photo-bioreactor designs^44^, upconversion nanoparticles^45^, or through tissue embedded probes in the case of medical applications^46^. Another limitation to the deployment of optogenetics is potential interference of optogenetic signals with endogenous light-responsive processes^47^. While limiting emitted wavelengths to those not perceived by the engineered organism could alleviate this issue, it might not be possible with current optogenetic technologies that are based on naturally occurring proteins. Using the tools of synthetic biology, it should be possible to engineer orthogonal light-responsive proteins or refactored host organisms to minimize off-target optogenetic effects. Further development of NIR chromophore proteins would ideally serve this purpose due to the limited absorbance of NIR radiation by biomass^48^. Another limitation to the implementation of heterologous optogenetic chromoproteins is the toxicity resulting from protein overexpression or interference with native physiology and phototoxicity. However, these can be overcome using protein engineering techniques, fine tuning light exposure, and presumably by lowering heterologous protein expression levels^49^.

It is difficult to predict future applications of optogenetics in bio-informational engineering. However, potential future applications could include ‘smart’ greenhouses where plant physiology is adjusted according to biosensor reported intracellular conditions using light^47^, light-controlled microbial patterning^50^ and biomaterial production^51^, or even to provide precise control over mammalian cells for smart-phone-mediated insulin release^52^, or for light-directed tissue engineering and developmental biology^53^. Finally, an intriguing future direction could be the development of ‘reverse optogenetics’ where NIR fluorescent proteins^54^ are used to report on specific components of intracellular physiology using biosensors. In contrast to traditional fluorescent proteins that emit visible light, NIR fluorescent proteins are easier to detect from deep tissues and could thus serve as a safe and rapid means of communicating intracellular conditions from tissue^54^.

### Bioelectrochemistry

Bioelectrochemistry is fundamental to all cells, which rely on ionic gradients to generate energy through ATP synthases, and reduction oxidation (redox) reactions to drive metabolism. Furthermore, there are naturally evolved specialized functions that harness electrochemistry, such as the neuronal action potentials that underlie brain function, bacteria that harness extracellular electrons, and ion and reactive oxygen species which mediate intercellular communication^5^.

The natural occurrence of bioelectrochemical signals has already enabled direct measurement and control of various biological signals and processes. For example, intracellular redox states have been perturbed to control the circadian rhythm independently of light in cyanobacteria using electrode-controlled cell permeable electron acceptors^55^. Plant wounding and herbivory is communicated in plants over long distances using calcium ion signalling and membrane potentials that can be both detected and stimulated using implanted electrodes^56,57^. Neuron-action potentials and inter-neuron neurotransmitter concentrations can be both measured and stimulated in living brains using electrodes^58^. Cellular redox in particular has proven to be a valuable source of cellular information that is easily accessible to human and device readable outputs. For example, detection and memory of redox state was achieved using a chatecol-chitosan-agarose matrix with optical or electrical output^59^. The ubiquitous nature of biological redox has made it possible to electronically interact with skin pigments such as melanin, control biological patterning and gene expression, and integrate two-way communication via redox stress signals^60^. However, the global nature of redox regulation in cells makes precise and modular use of this signal difficult in some circumstances. These technologies hint at a future where agriculture, human disease, and microbial biomanufacturing can be controlled and measured bio-electrochemically. However, the field lacks bio-informational engineering systems that are modular, orthogonal, and context independent.

### Challenges and future directions

#### Modular two-way bioelectrical communication using biosensors

The fields of synthetic biology and bio-electronics are converging to enable two-way bio-informational communication^61^. Synthetic biology is necessary for the engineering of generic architectures that can convert electrical signals into biochemical activity and vice versa. A prominent example of this type of bio-informational signal conversion is in the use of glucose dehydrogenases that can transfer an electron to an electrode in response to glucose levels (Fig. 2). The glucose dehydrogenase protein central to the sensor has since been engineered into a modular electron-donating biosensor architecture for measuring other molecules of interest. This modularity is achieved by splitting the protein and fusing it to LBDs (in Fig. 2) so that glucose dehydrogenase only forms its tertiary structure, and therefore its electron donating function, when the ligand of interest is present^25,62^. This type of modular biosensor engineering provides a conduit between biochemical and electrical information systems and has the extensibility necessary to allow for detection of many different biomolecules. Future deployment of this system will depend on the discovery or engineering of suitable LBDs to detect molecules of interest.

Generic architectures for electronic to biological signal conversion have also recently been developed by synthetic biologists. Electrodes were used to reduce cell permeable redox carriers (ferro/ferricyanide), which altered native *Escherichia coli* respiratory and redox processes to oxidize pyocyanin. Oxidized pyocyanin then induces transcription of genes of interest from the SoxS promoter by binding to the SoxR transcriptional regulator^63^. Electronic control of gene expression from the SoxS promoter was used to modulate diverse behaviours such as cell motility and quorum sensing. Because the SoxR/SoxS system could be expressed in other cell types that are supplied with redox carriers and electrodes, and can be used to control the expression of any genes of interest, this system represents the first generic electronic to biological converter. However, it is limited by the time delay for the gene expression output to occur (~45 min), by the specific interaction between supplied redox carriers and native respiratory components, and by the challenges in porting bacterial transcriptional regulation to eukaryotes. These limitations are not insurmountable, with many cells having respiratory systems that interact with ferro/ferricyanide, and emerging technologies for functional expression of bacterial transcriptional regulators in eukaryotes^19^. Additionally, if prokaryotic transcription factors are required for medical or diagnostic applications, they could simply be engineered into the host’s resident microbiome, where synthetic biologists have made significant progress recently^64^. The *E. coli* SoxR/SoxS bio-electrical system was subsequently improved using quorum-sensing-mediated signal amplification and noise dampening through native oxidative stress inhibition^65^. The fact that quorum-sensing behaviour was an electronically controllable output opens up the possibility of controlling microbial communities and microbiomes at larger scales. Despite the versatility and effectiveness of the *E. coli* SoxR system, it is not feasible to port to mammalian tissues due to genetic incompatibility and the toxicity of redox mediators. A major advance towards tissue compatible electrical induction of mammalian gene expression was recently achieved when human B-cells were engineered to release insulin in response to electrical stimulation^66^. Engineered cells have their resting membrane potential lowered via the expression of a potassium efflux protein so that electrical stimulus alters membrane potential to cause calcium ion influx through a voltage gated calcium channel. The signal is then transduced via the calcium responsive calmodulin/calcineurin pathway, which ultimately leads to activation of a transcription factor–promoter pair that drives expression and secretion of insulin.

A much needed advance in this field is the development of modular protein localization domains that can be coupled to electronic inputs, as exist in the field of optogenetics with light. Signal output that relies on protein–protein interactions or conformational changes rather than on transcription and translation can occur in seconds rather than minutes. One way to achieve this would be to express split redox cofactor-binding proteins that only co-localize in the presence of redox carriers in a particular state of reduction. In theory, this could be achieved using the interaction of SoxR with pyocyanin, where split SoxR is fused with other signal actuation domains. Alternatively, there is a pre-existing array of redox-sensitive fluorescent proteins such as HyperRed that could be co-opted for this purpose^67^.

The precise control afforded by existing electronics, the interfacing of electrical circuits and computers with biology, and the IoBT, is promising. One challenge in this vision is that the complexity of existing electronic circuits is not yet matched by the complexity of biological circuits, although there have been considerable advances made by the synthetic biology community, led by the Voigt lab^68–70^. If biological circuitry cannot match the complexity of electronic inputs, then there will be a sharp loss of information in the transition between optoelectronic and biological mediums. There are more than five decades differentiating the scientific and technological maturity of optoelectronic engineering versus bio-informational engineering^71^ . Bio-informational engineering will necessarily need to catch up with its optoelectronic and digital counterparts in order to ensure signal conversion between organic and inorganic information substrates does not result in information loss. Given the complexity of naturally evolved living systems, and the capacity to further build on this complexity^72^, it should be possible to design biological systems that efficiently integrate with optoelectronics and computing across the coming decades.

### Early adopters and potential applications

The development of integrated solutions enabling biology-to-digital information pathways are edging closer to real-world applications. For example, Indigo Ag develops microbial treatments for crops, but in 2018 they purchased the satellite imaging company TellusLabs. The two companies noted that this merger brings together datasets that can be leveraged via machine learning to better target products to individual farms. From a bio-informational perspective, TellusLabs has begun acquiring the different types of multiscale functionality required to develop and deploy persistent environmental monitoring networks. Digital-to-biology control loops are also under active investigation and testing. The company Berkeley Lights is a good example of this; they use light to automatically move individual cells into discrete, nanolitre-sized chambers that are ~100,000 times smaller than a microwell. As another example, the company Aromyx designs olfactory biosensor arrays for the detection of aqueous and gaseous substances, combining bioengineered yeast and mammalian cell platforms with luminescence, fluorescence, and colorimetric output. Engineering organisms that respond in a well-characterized manner to optical signalling will enable digital-to-biology control loops with low latency. Optogenetics and bioelectrochemistry are opening up novel ways to control cell behaviours, and integrating biology with two-way digital control loops further expands the vast design space of biological devices.

#### Multiscale biofoundries

**Fig. 3:**
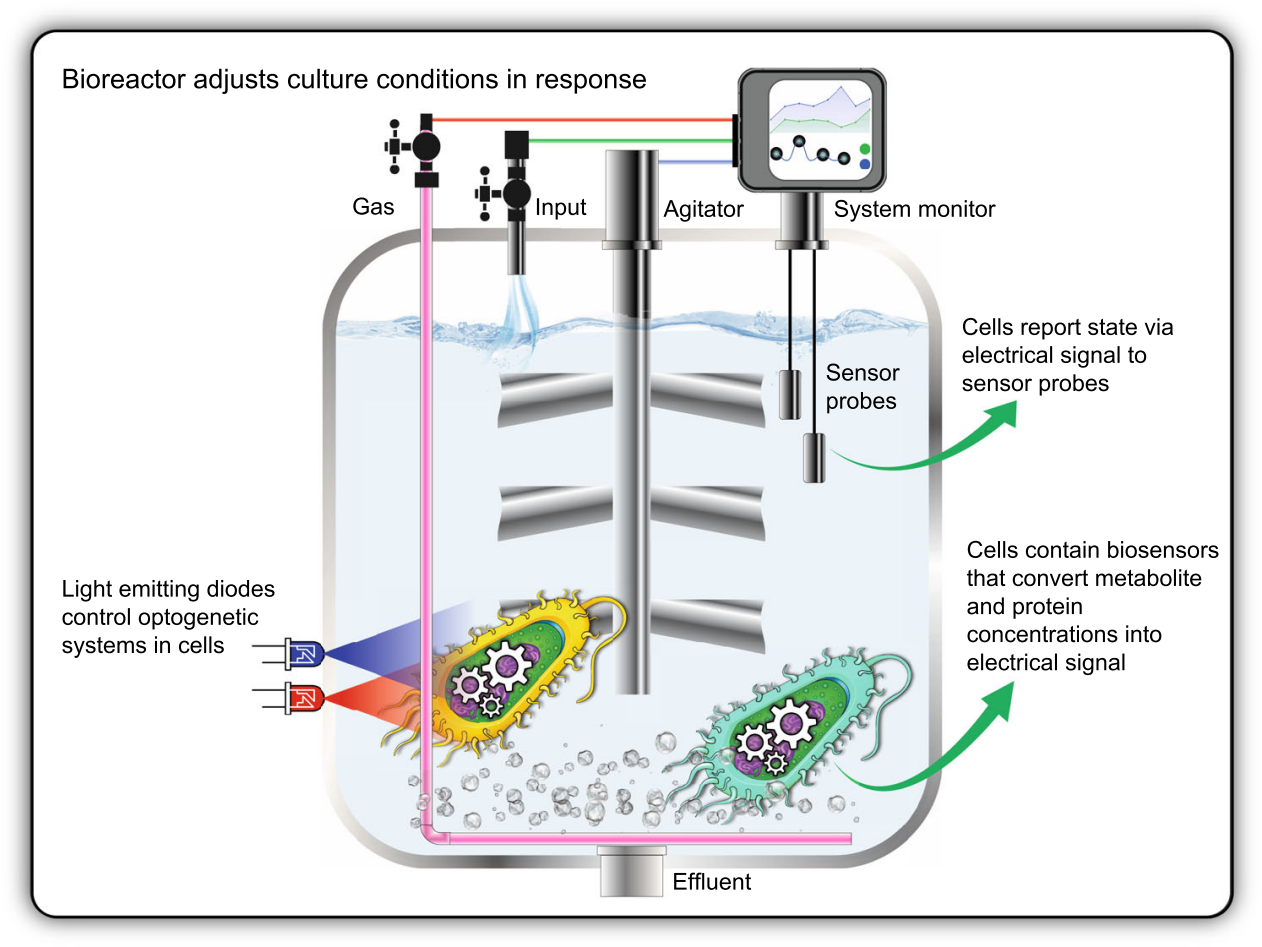
An autonomous bioreactor enabling smart culture condition control and the targeted actuation of engineered gene expression without human intervention. Bio-informational systems involving optogenetic control of gene expression and biosensor-mediated output of intracellular physiological states could be integrated within a bioreactor to facilitate high-yield production of sustainable chemicals, fuels, foods, materials, and pharmaceuticals using microbial cell factories.

Biofoundries stand to gain significant productivity benefits from further automating the *design-build-test-learn* (DBTL) cycle. Research biofoundries might become testbeds for biosensing soft robotics and autonomous bioreactors (Fig. 3), experimenting with the integration of autonomous and chemically aware control loops throughout their DBTL workflows. Biosensors are likely to have an immediate impact during the *Build* and *Test* phase of biofoundry-mediated engineering, where intracellular metabolite or protein levels can be actuated into a fluorescence, electrical, or survival response to enable screening of millions of genetic variants per day rather than thousands^15,73,74^. This would complement already existing routes for automation, such as image recognition approaches to monitoring combinatorial design at the macroscopic level. Importantly, pattern recognition has been flagged by the Engineering Biology Research Consortium (EBRC) as key to developing the mature computational infrastructure needed to support next-generation biodesign^74^. After superior strains are identified, their use in industrial-scale biomanufacturing could also be optimized using bio-informational control loops in autonomous bioreactors. Just as traditional bioreactors monitor the production of carbon dioxide and the consumption of oxygen by microbial metabolism and automatically trigger carbon source feeding when these profiles change, bio-informationally integrated bioreactors could adjust culture conditions or cellular physiology in response to biosensor-mediated monitoring of intracellular metabolite or protein concentrations. Biosensor constrained host cell physiology is an emerging technique in metabolic engineering that has been used to optimize fatty acid and tyrosine production in *E. coli*^15^. Economically viable cell-length bio-manufacturing would complement existing commercial solutions for enzyme-length bio-manufacturing that are provided by companies like EngineZyme, Codexis, Arzeda, Zymergen, Amyris, Ginkgo Bioworks, LanzaTech, and Calysta.

The potential of bio-informational engineering integrated biofoundries and bioreactors as general-purpose bio-manufacturing platforms hints at the potential of the bioeconomy and the emerging circular economy. There is great economic potential to be found in harnessing carbon-waste streams as a feedstock for bio-manufacturing. The recent conversion of *E. coli* and *Pichia pastoris* to generate all biomass carbon from carbon dioxide indicates the adaptability of workhorse model organisms in relation to directed evolution design strategies^75,76^. These metabolic rewiring and directed evolution projects indicate that chassis organisms are likely to be optimized for industrial carbon fixation (for long- and short-lived purposes) in biofoundries (for DBTL) and autonomous bioreactors (for industrial scale-up). Next-generation bio-manufacturing will in part rely on the capability of waste carbon reuse^77^, and this will be enhanced through bio-manufacturing platforms that utilize optogenetic and bioelectrical control functions to improve production titres, rates, and yields. This will be an important route for industrially scaling economically competitive carbon-neutral and carbon-negative manufacturing processes. The success (or not) of bio-informational deployments in next-generation biofoundries would provide commercial proof-of-concept for first-generation bio-informational engineering. This ‘first adopter’ status means biofoundries are likely to form those communities that will first encounter and need to negotiate bio-informational ethical, legal and social implications (ELSI). The critical next step for biofoundries is therefore the exploration, and alignment, of their activities with community expectations and confidence levels.

#### Precision agriculture and environmental monitoring

**Fig. 4:**
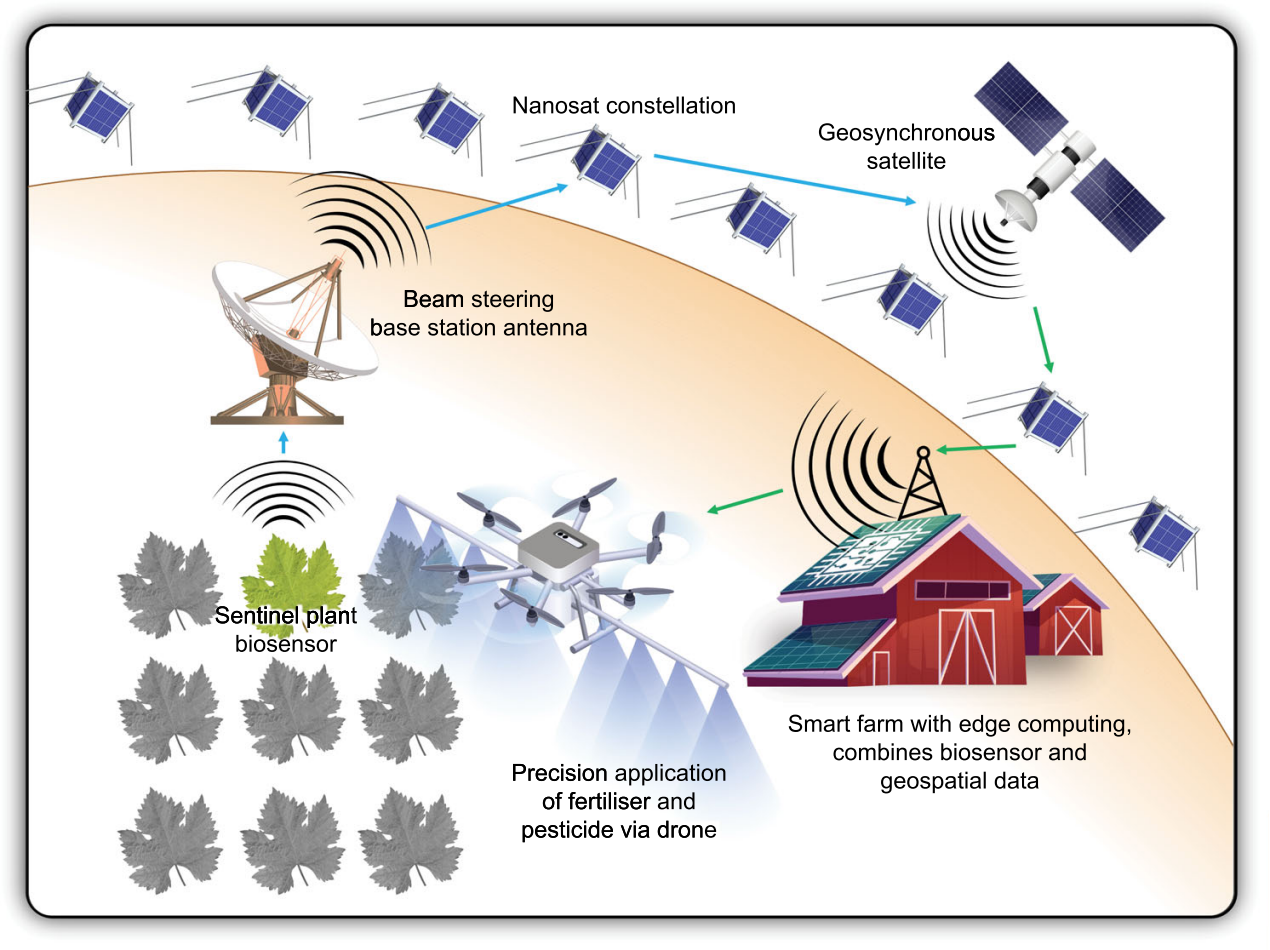
A sentinel plant-enabled precision agriculture control loop integrating space-based capability. Harvested sentinel plant sensor signals could be communicated to a proximal smart farm through an Internet of Biological Things satellite architecture in real-time with low latency. Edge computing at the smart far could combine sentinel signals with daily-refreshed geospatial and current local weather forecasts. Embedded artificial intelligence agents at the smart farm could undertake insight-driven drone route planning to optimize autonomous application of fertilizer and pesticide.

In agriculture, crop-monitoring control loops could be linked to drone surveillance route planning, facilitating the autonomous investigation of crops experiencing heat, water, or disease stress while triggering targeted nitrogen or phosphate release through two-way bio-informational communication. The integration of sentinel crop biosensors^78^ with nanosat constellations and cloud or edge computing architectures could enable the remote monitoring and precision management of agriculture. Farm management could be more targeted, more sensitive and apply machine learning generated insights arising from the analysis of other data streams. Data integrations could include historical weather data, short- and long-term forecasting, as well as geospatial imagery with daily refresh rates (e.g. imagery from the company Planet or Indigo Ag). Figure 4 provides an example of a multiscale agricultural monitoring and control network.

Similarly, in environmental monitoring, a spike in local pollution levels monitored by a biosensor^10^ network could trigger the re-pointing of nearby high-resolution satellites for follow-up investigation before a human is needed to enter the decision-making loop. By substituting mine infrastructure for the crop plantation in Fig. 4, it is easy to imagine extensible applications for multiscale living monitoring networks. Environmental pollution could be monitored through biosensor deployments surrounding tailings storage. Coal seam gas extraction could be monitored across wide areas of agricultural and wild landscape. Many statutory environmental monitoring protocols could be automated through networked biosensor deployments that integrate artificial intelligence data handling.

Plant responses to pathogens have shown they can be linked to a single dominant gene^79^. However, the role of salicylic acid is critical to regulating many aspects of plant growth, development, and defense. It has become increasingly apparent that salicylic acid does not signal immune responses by itself, but is part of an intricate network that involves many other plant hormones^80^. Though there is much work to be done on characterizing plant immune responses^81^, it does suggest that transgenic plants could be engineered to have heightened responses to specific pathogens through promoting defense-signalling mechanisms. Plants are able to effectively cope with invading pathogens by activating an immune response based on the detection of invasion patterns originating from the pathogen or released by the plant after infection^82^. Plants in proximity to high-risk locations (like markets, farms, and feedlots) could be engineered to monitor for animal-borne pathogens for which there is a known risk of zoonotic transfer. In particular, abattoirs provide a well-suited monitoring location as pathogens would be more easily detected by interfacing sentinel plants with slaughterhouse water runoff. While this would not be the simplest route for monitoring well-characterized animal-borne pathogens—target-molecule monitoring in animals would likely be more economical—it would offer a unique avenue for monitoring unknown animal-borne pathogens (Disease X) that have yet to be evaluated for their zoonotic threat potential. By deploying plants that would normally be proximal to animals in wild environments, the natural defenses of those plants to herbivore-borne pathogens could be monitored. This could be transformative for animal husbandry by enabling emerging infectious disease monitoring of unknown pre-zoonotic pathogens via plant-based living systems. By connecting digitally enabled target molecule monitors to sentinel plants in slaughterhouse runoff, an IoBT approach could be used whereby the artificial intelligence monitoring of incoming plant-based signalling is used to identify Disease X encounters.

The integration of target molecule monitors (Fig. 2) with natural plants is likely to encounter lower levels of regulatory review than integrating target molecule monitors with engineered transgenic plants. Monitoring natural plant responses to pre-zoonotic high-risk animal-borne pathogens may provide a novel approach to the persistent monitoring of emerging infectious disease prevalence among animal populations. Given the recent history of zoonotic transfer^83^ and the impact this can have socially, economically, and culturally on human communities, novel techniques for monitoring and managing high-risk and unknown zoonosis are likely to be investigated across the coming years. Across the longer term, one could expect that natural and transgenic sentinel plants may be optimized for a range of persistent monitoring deployments that enhance precision agriculture, environmental monitoring, and emerging infectious disease early warning systems.

#### Precision medicine and plug-and-play applications

**Fig. 5:**
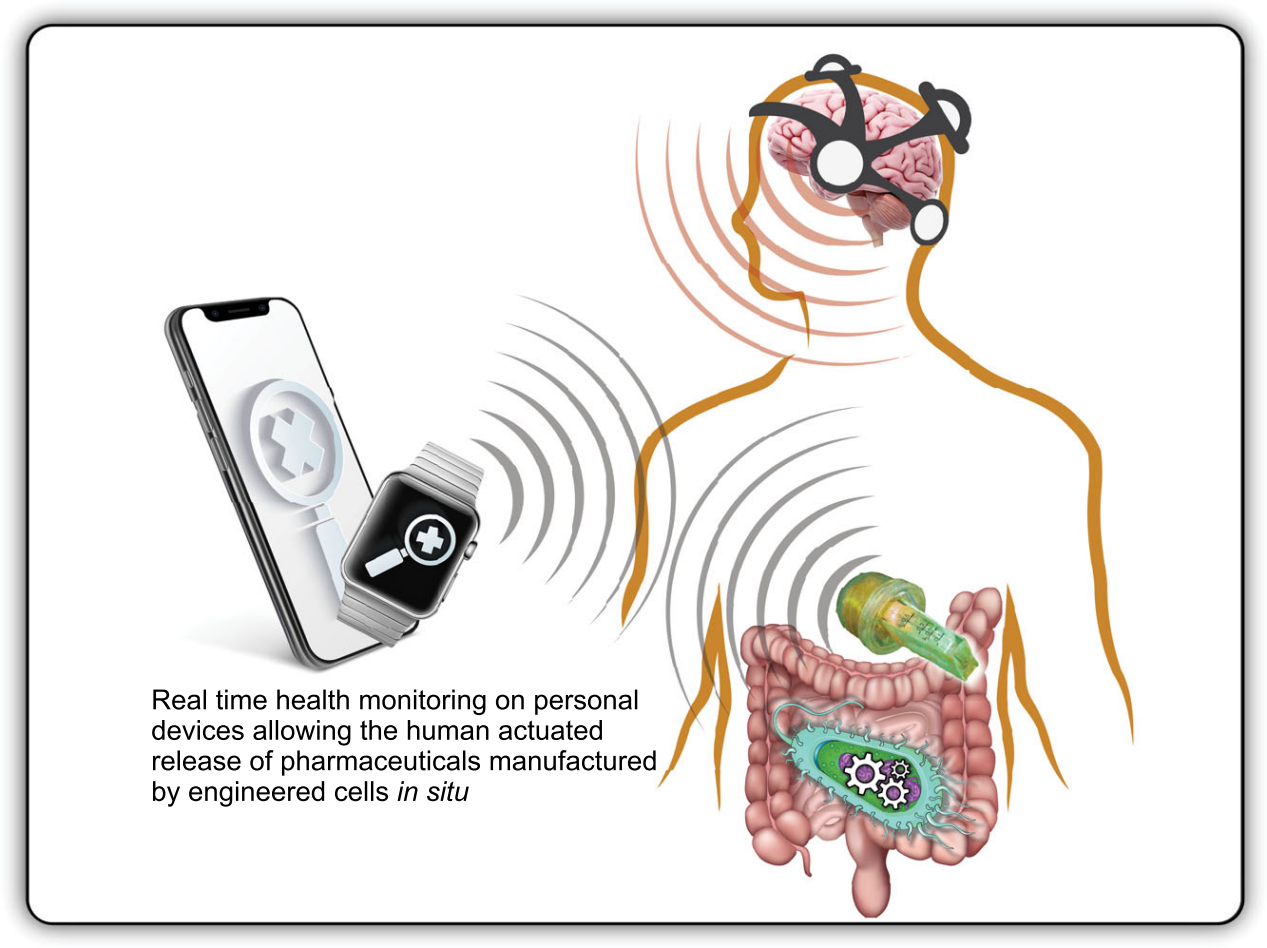
Optogenetic designer cell implants bio-informationally integrated with wearables, personal devices, and EEG-based actuation for specific use cases. Longer-term applications include viewing health information on a personal device reported via a subcutaneous optogenetic device with bioelectrical monitoring properties. Engineered microbiomes could both report real-time patient data and deliver on-demand pharmaceuticals in situ.

An optogenetic or bioelectrochemical pathway that links the chemical outputs of a natural or synthetic organism with digitally interoperable control loops has a wider range of potential applications than just environmental monitoring. In health, deployments could include medical devices designed for human in situ use, and two-way controls are likely to be particularly beneficial in biomedical applications. Over time, this could allow for the controlled release of chemicals from engineered gut microbiota to be integrated with wearable and smart phone technologies. In the longer term, this could supplement the oral ingestion of medicines (particularly for paralyzed patients) as well as ensure a patient’s medicine levels are more carefully calibrated to their needs through the real-time monitoring of bodily fluids via biosensors^84,85^. Figure 5 provides an overview of the signal communication points currently under investigation. It does not escape our notice that a 2016 proof-of-concept study linked the human use of electroencephalographical (EEG) headsets into a bio-informational control loop actuating optogenetic-linked chemical release in mouse models^84^. The integration of mental-state specific brain wave activity with bio-informational control loops promises a near infinite variety of potential use cases both in and outside the biomedical domain. One of these being novel research that correlates phenomenological and phenotypic events for organisms with neural substrates, including humans. The diversity of possible use cases indicates a horizon of potential in which synthetic cells make promising candidates as subcomponents within integrated, flexible and bioinspired structures, such as soft robotics, wearables, and subcutaneous technologies. The company Neuralink is a good example of commercial research and development in this space.

There now exists an opportunity to create functionally useful microbiomes for soft robotic substrates through harnessing optogenetic pathways of information exchange. This indicates an emerging world of plug-and-play IoBT appliances that would create industrially useful capabilities across many different commercial sectors. While much work in optogenetics has been focused on applications related to neural substrates, advances made in relation to soft, stretchable, and fully implantable miniaturized systems^86^ are promising. These include prosthetic electronic skin^87^ to interface with natural and engineered biology, and plastic bioelectronics that take advantage of the nano-scale miniaturization of silicon chips^88^. Over time, this may lead to an increased reliance on organic electrochemical transistors^89^, including printed logic circuitry and neuromorphic devices. These organic devices may be more suitable for biological interfacing than inorganic alternatives.

Bio-informational plug-and-play devices are approaching a barrier that has traditionally walled off two-way communication between biological and digital systems. This is in part due to the analytical capacities of artificial intelligence and the role such algorithms can play in extricating information from high levels of signal noise. Artificial intelligence data handling will very likely be an essential enabler of bio-informational engineering due to its algorithmic capacity for automating decisions and generating meaningful signals based on big data pattern analysis^90^. This is a keystone capability for engineering bio-informational control loops that involve noisy signal actuation, monitoring and translation across organic and inorganic information substrates. At smaller length scales, AI is already helping to fine tune and predict synthetic biological designs. This was recently demonstrated through machine-learning-mediated gene expression engineering^91,92^ and AI-assisted rational metabolic engineering^93^. AI-enabled bio-informational devices are likely to disrupt current assumptions for how communication systems work in relation to biological processes and biological information management. We fully acknowledge the disciplinary and industrial terms *biology* and *information* have clear and distinct bounded definitions today. However, just as bioinspired devices are dissolving two-way biology-to-digital communication barriers, we anticipate the disciplinary borders of the biological and information sciences will become more difficult to rigidly define over the coming decades. This is a potential boon to many economic sectors, domains of health and medicine, and potentially of great benefit to the environment. Yet, it represents a distinct change in the way living organisms interact with engineered systems. A multiscale taxonomy for describing these engineering objectives is going to be increasingly important. It will matter not whether a system is biological or digital, organic or inorganic, but rather, it will matter which length scales the system is engineered across and what information substrates are used. The interfacing of chemical, electrical, and optical information substrates across bio-informational systems will transform ideas of what information is, where it is stored, and how it is structured. Perhaps more importantly, these capabilities are maturing at a time when the most disruptive biological event in a century is playing out across the world stage.

### A cautiously optimistic outlook

The COVID-19 pandemic is significantly shifting both the expectation and confidence levels of governments and publics around the world. Across the long term, this may result in an increase or decrease of trust in the capabilities being developed by engineering biology. It remains unclear how those publics that have traditionally taken an oppositional stance to engineering biology will respond to treatments and vaccines that are a product of that discipline and practice. This may have implications for how governments undertake COVID-19-related public health interventions, and whether or not those interventions are successful^94^. Governments around the world will be searching for technologies that can monitor the environment and integrate information flows with public health early warning and response systems. This is likely to incorporate traditional technological solutions in the age of big data—i.e. artificial intelligence data handling and insight generation^90^—but it also suggests the outlook for novel bio-informational solutions is one of increased public and private funding. In order to maintain legitimacy and support for population-level bio-surveillance tools across the long-term, governments will need to undertake nuanced and sophisticated communication programs. Anti-vaccination sentiment could be a forerunner for oppositional sentiment to multiscale bio-informational monitoring via environmental surveillance systems and the IoBT. It will be critical from the outset that governments communicate the value of these programs in ways that bring all publics along^95^. The impact on biofoundries and next-generation bio-manufacturing platforms arising from poorly executed science and public health communication is likely to be acute. It is probable that biofoundries may transition into standing pandemic response platforms that support the wider bioeconomy when they are not deployed for emergency response. But this will only occur if the underpinning technologies maintain the legitimacy and support of government, industry, and the community. The response of these actors to developing pandemic preparedness capabilities will be an important area to watch. Whether or not countries take up the opportunity of developing and maintaining pandemic response bio-manufacturing platforms will ultimately be dictated by their national politics. The emerging bioeconomy could be enabled or disabled by the shockwaves of COVID-19, and this will necessarily have an impact on the future of bio-informational technology research and development.

To date there has been significant investment in rapid vaccine development and virus-testing capabilities. If political legitimacy for emerging infectious disease public health interventions is maintained, a post-pandemic world could be one of pervasive and persistent environmental monitoring in urban, rural, and wild environments. The only difference to current forms of monitoring being the addition of digitally enabled biosensors. An important consideration for those that design, build, test, and scale-up bio-informational engineering solutions will be that of information security risks. Cyber security risks are unlikely to remain localized to electrical and inorganic cyber-physical systems during a period of growth for bio-informational applications in precision medicine, precision agriculture, and bio-manufacturing^96^. The practice of cyber security will need to be thought of more generally as the management and mitigation of information security risks, regardless of an underpinning system’s engineering length scales or the material composition of a system’s information substrates. The post-COVID balancing act of biosecurity versus bio-privacy has already come into sharp relief. The race for a vaccine has seen an increase in cyber-attacks on genomic, health care, and medical data stores worldwide. The future of multiscale bio-informational engineering is just one of many areas that will require new responses from governments, industries, and communities in the coming years. The potential and novelty of two-way bio-informational communication necessitates a unique approach that balances the competing needs of all stakeholders. This is an area that would benefit from investigation and analysis by STEM and HASS scholars alike, ideally in collaboration with each other as well as practitioners and policy makers.

Practitioners, technicians, and scientists cannot afford to be anything but open and transparent about the potential of bio-informational engineering. The 2020 memetic propagation of the COVID-5G telecommunications myth is just one example of how publics can drastically overestimate scientific and technological capability to the detriment of informed public debate. These types of misinformation events should not be met with derision from the scientific community, but instead require respectful and engaged two-way communication with those publics that take negotiated or oppositional stances on the benefits of engineering biology. This is not an easy thing to do, but it will ensure that bio-informational science and technology remains governed via democratic deliberation as noted by the Presidential Commission that examined synthetic biology in 2010 (ref. ^97^). Responsible innovation and an open approach to benefit sharing must be buttressed by public legitimacy for scientific and technological development objectives. Due to the capacity for bio-informational engineering to significantly widen the biological design space open to humanity, it is recommended that responsible innovation practices be continually evaluated in relation to novel bio-informational use cases as and when new use cases arise. With this in mind, we maintain a cautiously optimistic outlook for the future of bio-informational engineering. As national and international regulatory regimes^97^ adapt to COVID, they will lay the groundwork for regulating the novelty of multiscale bio-informational engineering. The extension of synthetic biology, systems biology, and engineering biology into new design and solution spaces will be a transformative development. It is a development we look forward to contributing to.
